# Synthesis of Porous BPPO-Based Anion Exchange Membranes for Acid Recovery via Diffusion Dialysis

**DOI:** 10.3390/membranes12010095

**Published:** 2022-01-16

**Authors:** Muhammad Imran Khan, Abdallah Shanableh, Majeda Khraisheh, Fares AlMomani

**Affiliations:** 1 Research Institute of Sciences and Engineering (RISE), University of Sharjah, Sharjah 27272, United Arab Emirates; raoimranishaq@gmail.com (M.I.K.); shanableh@sharjah.ac.ae (A.S.); 2 Department of Chemical Engineering, College of Engineering, Qatar University, Doha P.O. Box 2713, Qatar; falmomani@qu.edu.qa

**Keywords:** BPPO, diffusion dialysis, trimethylamine, acid recovery, porous anion exchange membrane (AEM)

## Abstract

Diffusion dialysis (DD) is an anion exchange membrane-based functional separation process used for acid recovery. TMA (trimethylamine) and BPPO (brominated poly(2,6-dimethyl-1,4-phenylene oxide) were utilized in this manuscript to formulate AEMs (anion exchange membranes) for DD (diffusion dialysis) using the phase-inversion technique. FTIR (Fourier transfer infrared) analysis, proton NMR spectroscopy, morphology, IEC (ion exchange capacity), *LER* (linear expansion ratio), *C_R_* (fixed group concentration), *W_R_* (water uptake/adsorption), water contact angle, chemical, and thermal stability, were all used to evaluate the prepared membranes. The effect of TMA content within the membrane matrix on acid recovery was also briefly discussed. It was reported that porous AEMs have a *W_R_* of 149.6% to 233.8%, IEC (ion exchange capacity) of 0.71 to 1.43 mmol/g, *C_R_* (fixed group concentration) that ranged from 0.0046 mol/L to 0.0056 mol/L, *LER* of 3.88% to 9.23%, and a water contact angle of 33.10° to 78.58°. The *U_H_* (acid dialysis coefficients) for designed porous membranes were found to be 0.0043 to 0.012 m/h, with separation factors (*S*) ranging from 13.14 to 32.87 at the temperature of 25 °C. These observations are comparable to those found in the DF-120B commercial membrane with *U_H_* of 0.004 m/h and S of 24.3 m/h at the same temperature (25 °C). This porous membranes proposed in this paper are excellent choices for acid recovery through the diffusion dialysis process.

## 1. Introduction

Metal etching, pickling, and stripling processes generate a lot of waste solutions containing inorganic acids [1,2,3,4,5], whereas organic acid-containing waste solutions are created by fermentation, food, leather, and pharmaceutical corporations [1,2,6,7,8]. Different processes, such as neutralization [9,10,11], coagulation and flocculation [12], extraction [13], and the ion exchange process [14,15,16,17,18,19], can be used to recover the acids. In the chemical and biochemical sectors, ion exchange membranes (IEMs) play an integral role [20]. Ion exchange membranes (IEMs) that are delicate films (usually very thin) composed of immobile positively or negatively charged functional groups and counter ions are gaining popularity since they make separation processes simpler, more productive, and less expensive. AEMs (anion exchange membranes) and CEMs (cation exchange membranes) are two major categories of IEMs. AEMs have lately attracted considerable attention because they can effectively retrieve acid wastes from various industries such as mining, metal manufacturing, painting, and agriculture [21,22,23,24].

**Figure 1:**
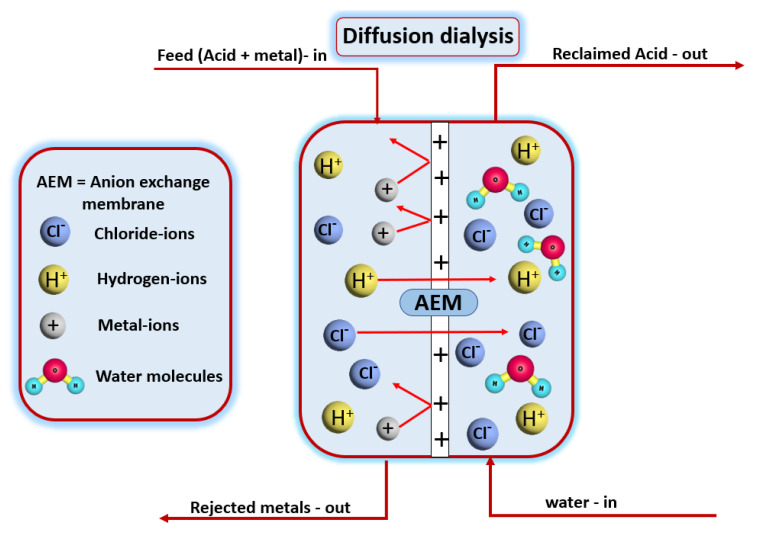
Graphical representation of the diffusion dialysis process through the anion exchange membrane separation process of HCl from its inlet feed solution.

The DD (diffusion dialysis) process is a versatile strategy that utilizes (AEMs) anion exchange membranes for the extraction and recovery of acids [25,26,27,28,29,30,31]. To be more specific, the diffusion dialysis process depends on a concentration gradient for removing and purifying the acid or alkali waste solutions using the ion-exchange membrane separation method [8,23]. High energy savings [32,33], environmental safety [17], and continuous operation [34] are all advantages of the DD process. When producing chemicals, the acids can be recycled and reused, whereas the waste solution’s residual products, such as metal ions, can be eliminated immediately [8]. The diffusion dialysis mechanism is shown in Figure 1 by the separation of HCl from its inlet solution.

**Figure 2:**
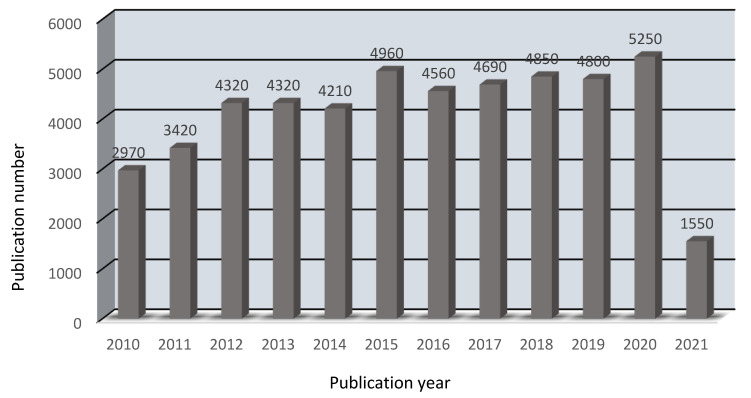
A brief post-2010 timeline depicting the number of associated academic publications for the acid retrieved through diffusion dialysis process with anion exchange membranes (Available online: Google Scholar (12 December 2021).

A concentration gradient exists between the feed and outlet sides, as seen in Figure 1. As a result, both the hydrogen ions (cations) and the chloride ions (anions) diffuse across the anion exchange membrane. Although being positively charged, hydrogen ions are capable of passing across the AEM due to their compact size, high mobility, and lower valence state. Metals in the feed solution do not pass through the AEM, so they are rejected. From the outlet side, the HCl is extracted. As a result, the acid is successfully extracted from the inlet solution. The use of AEM to recover acid through diffusion dialysis has been extensively proposed and applied around the world. The number of publications per year in this area are shown below in Figure 2.

There is a general upward trend in the number of publications each year, as seen in Figure 2. This demonstrates that acid recovery through the diffusion dialysis process using an AEM has received a lot of attention in the last decade.

Because of their prominence in DD, anion exchange membranes have received considerable attraction [23,26,27,28,31,35]. Polymers such as PS (Polystyrene), PSf (Polysulfone), and BPPO (brominated poly(2,6-dimethyl 1,4-phenylene oxide)) make up the bulk of homogenous acid recovery AEMs [8]. Presently, the dense membranes make up the plurality of AEMs used in acid recovery via diffusion dialysis [36]. Direct evaporation is used to typically manufacture the polymers’ solution (homogeneous membrane) such as poly(p-phenylene oxide), polystyrene, and polysulfone [8], or the copolymerization of monomer units within the pores of a porous membrane surface that has already been formed (heterogeneous membrane) [37,38,39,40]. They are, however, often thick and compact (several tens or hundreds of micrometers), which can obstruct the transportation of ions into the membrane [8]. The acid dialysis coefficient (*U_H_*) of the BPPO-based commercial membrane DF-120B, for instance, is indeed just 0.004 m/h at 25 °C [41]. Due to poor permeation concerns, this study centered on incorporating a new feature into the membrane matrix. The starting material was chosen to be polymers that are hydrophilic in nature, typically polyvinyl alcohol (PVA), and the assistant functional groups of –Si–OH and –C–OH were integrated onto it [42,43]. At 20 °C, the membranes enhanced their *U_H_* values from 0.007 m/h to 0.015 m/h. [43]; however, the key disadvantage of the membranes based on PVA was that they swelled a lot in the water, making their long-term stability a concern. To address these difficulties, tiny alkoxy silanes such as tetraethoxysilane (TEOS) or multi silicon copolymer forming groups were used to functionalize and cross link the PVA main chains [43,44]. When we switched from dense membrane to porous membrane processing, we had to change our strategy to solve these issues.

**Figure 3:**
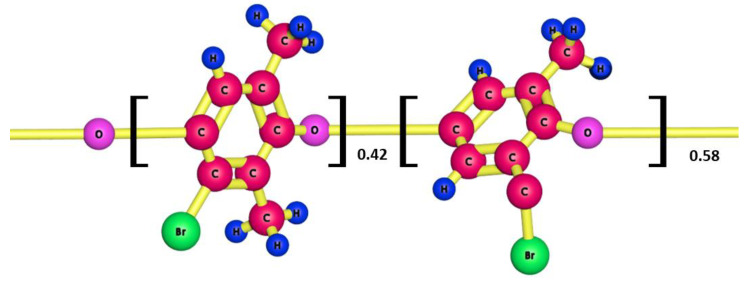
A three dimensional (3D) structure of brominated poly(2,6-dimethyl 1,4-phenylene oxide) (BPPO).

BPPO was chosen as the polymer matrix since the feasibility of making a porous membrane out of a hydrophobic polymer could be established. Figure 3 below depict the three-dimensional structure of the BPPO:

We recognize that the porous structure lowers ion transport resistance, whereas the polymer increases membrane durability. Apart from DD, porous membranes are frequently used in several different methods. These membranes, for example, are typically used in membrane processes that depend on pressure gradients such as ultrafiltration and nanofiltration. Moreover, for desalination purposes, to fabricate porous composite ion-exchange membranes, other research teams employed a two-step approach of phase-inversion [45,46]. Despite the fact that these micropores do not penetrate through the entire membrane, they can help HCl diffusion greatly [47].

**Figure 4:**
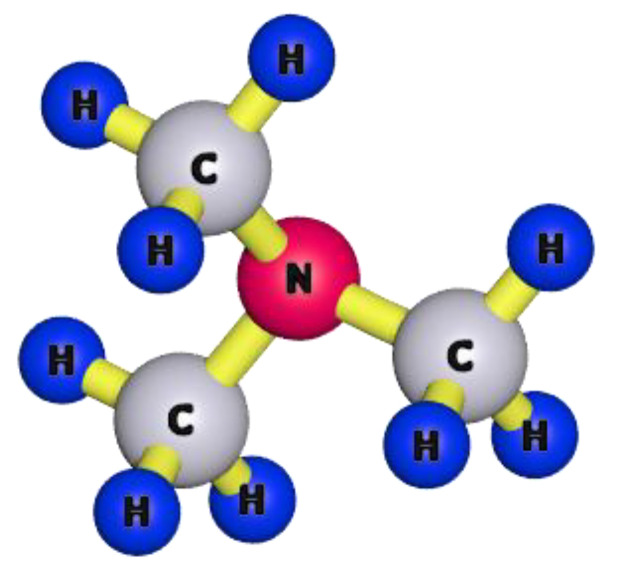
Three dimensional (3D) structure of Trimethylamine (TMA).

This paper describes the phase-inversion process used to formulate and characterize BPPO-based porous anion exchange membranes in ethanol medium using the reaction of BPPO and TMA. By varying the concentration for TMA (Figure 4) into the membrane matrix, the porous AEMs have been developed with different physicochemical characterizations. In batch mode, they were compared to the commercial DF-120B membrane for acid recovery through the DD process.

## 2. Experimental

### 2.1. Materials

Tianwei Membrane Co. Ltd., Shandong, China, provided brominated poly(2,6-dimethyl-1,4-phenylene oxide) (BPPO) and an anion exchange membrane that is commercially available; DF-120B (part of the DF-120 series). DF-120B is composed primarily of quaternized poly(2,6-dimethyl-1,4-phenylene oxide) (QPPO) and polyester as a substrate. Typical characteristics of this membrane include water uptake of 74.2% with IEC of 0.83 mmol/g. Trimethylamine (TMA), ethanol, hydrochloric acid (HCl), ferrous chloride (FeCl_2_·4H_2_O), sodium chloride, potassium permanganate (KMnO_4_), and sodium carbonate (Na_2_CO_3_) were obtained from Sinopharm Chemical Reagent Co. Ltd., China. The rest of the reagents used in the experiments were of analytical grade and commercially available from domestic chemical reagent manufacturers. These reagents were used without further purification. In addition, throughout the experiment, deionized water (DI water) was used.

### 2.2. Preparation of Porous Membranes Based on BPPO

**Figure 5:**
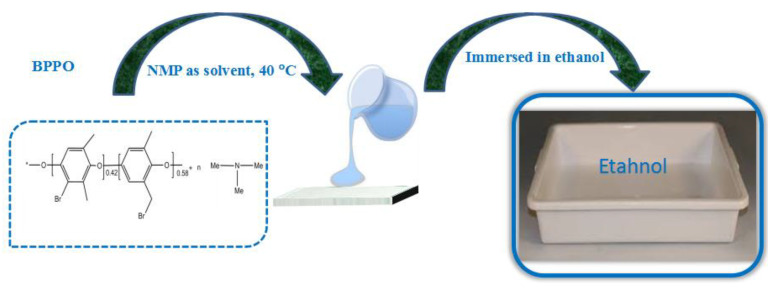
Illustration of phase-inversion method for the fabrication of anion exchange membrane.

**Figure 6:**
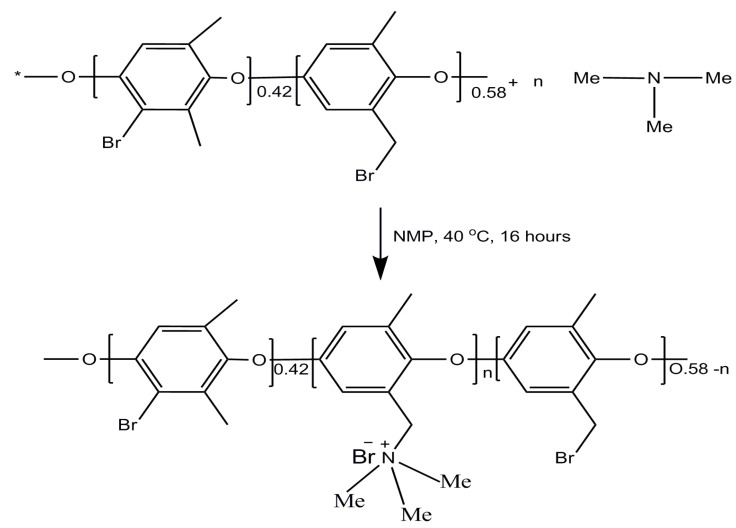
Designing of anion exchange membranes based on BPPO.

The procedure of phase-inversion was used for the fabrication of the porous AEMs, as mentioned previously in our studies [41,48]. Firstly, 3 g of BPPO (brominated poly(2,6-dimethyl-1,4-phenylene oxide)) was dissolved into NMP (N-Methyl-2-pyrrolidone) solvent at ambient temperature in a typical procedure. To obtain porous AEMs with different physicochemical properties, different amounts of trimethylamine (TMA) varying from 0.15 to 0.30 g were applied to the casting solution according to Table 1. The reaction mixture was stirred overnight at 40 °C to accelerate the reaction between BPPO and trimethylamine. The solution was subsequently poured onto a glass plate and immediately immersed in an ethanol medium (Figure 5). The membranes were cleaned by infusing them in water for two days straight, consecutively. The membranes were given the labels M1, M2, M3, and M4 according to the quantity of amine utilized. The formulated BPPO-based membrane’s chemical composition is also shown in Figure 6.

### 2.3. Characterizations

#### 2.3.1. Instrumentations

Using an FTIR spectrometer (Vector 22, Bruker, Billerica, MA, USA) with a resolution of 2 cm^−1^ and a total spectral range of 4000–400 cm^−1^, the attenuated total reflectance (ATR) technique was used to record the FTIR spectra for the dry membranes. The chemical structures of the BPPO and prepared membranes were also identified using a proton NMR (DMX 300 NMR spectrometer operating at 300 MHZ). With a Shimadzu TGA-50H analyzer, TGA was conducted on the developed membranes in the range of temperatures from 25 °C to 700 °C under nitrogen flow and a heating rate of 10 °C/min. The structure or morphology of the membrane was examined using a field emission scanning electron microscope (FE-SEM, Sirion200, FEI Company, Hillsboro, OR, USA). Dry membranes were analyzed with their surface and cross-sectional views. As representative cases, SEM illustrations of various porous BPPO-based membranes were displayed. For ease of handling, the formed membranes’ water contact angle was calculated at room temperature. A sessile drop method was used to obtain contact angle values from a one-contact-angle goniometer (SL200B, Shanghai, China) configured with video capture. In total, 40 μL of water was sprayed onto a membrane that was dried using a micro-syringe in a concentrated water vapor environment.

#### 2.3.2. Ion Exchange Capacity

The ion exchange capacity (IEC) of AEMs is indeed a significant parameter that determines how well they function in diffusion dialysis. In reality, AEM should prioritize high ion exchange capacity to improve ion transport sites [23]. IEC is a membrane permeability parameter that is dependent on the functional groups in the membrane matrix [49,50]. It is the amount of exchangeable ionic groups (equivalents) available per dry membrane weight. The traditional Mohr approach was used to measure it [12,51]. To ensure that all charge sites were transitioned to the Cl^−^ form, the samples of the membrane were stabilized in 1.0 (M) NaCl solution for 2 days. To remove any remaining NaCl, the membranes were then cleaned thoroughly with deionized water. After that, the membranes were stabilized for two days with Na_2_SO_4_ solution with a concentration of 0.5 (M). Titration was carried out with a concentration of 0.05 (M) AgNO_3_ and K_2_CrO_4_ as an indicator which was used to predict the quantity of Cl^−^ ions that were released. Furthermore, the membrane’s IEC (ion-exchange capacity; mmol/g) was determined using the formula, IEC = VC/m, where m, V, and C signify the membrane’s dry weight, titre volume during titration, and AgNO_3_ solution concentration, respectively.

#### 2.3.3. Water Uptake, Linear Expansion Ratio, and Fixed Group Concentration

*W_R_* (Water uptake) measurements were utilized to explore the membrane’s hydrophilic nature. To validate the dry weight for the samples of the membrane, they were heated and dried in an oven and weighed precisely. After that, for 72 h, these membranes were submerged in water at a temperature of 25 °C, and their wet weight was recorded accordingly after the water from the surface of these membranes was removed using tissue paper. The *W_R_* values were then determined using the mass difference before and after the membranes were entirely dried [41,52] using the following Equation (1) below; as the relative weight gained per gram for the dried sample:

(1) $$W_{R}=\frac{W_{WET}-W_{DRY}}{W_{DRY}}\times 100\%$$

where *W_DRY_* and *W_WET_* denote the weights of dry and wet membranes, respectively.

The dry and wet length data of membranes at room temperature were used to calculate the linear expansion ratio (*LER*). The following equation was used to determine the *LER* of prepared AEMs [53,54].

(2) $$LER=\frac{L_{w}{}_{}-L_{d}}{L_{d}}\times 100\%$$

where the dry and wet membrane lengths, accordingly, are denoted by *L_d_* and *L_w_*.

According to our prior studies, the fixed group concentration in the membrane was determined using (*W_R_*) water uptake and (IEC) ion exchange capacity [41,48].

#### 2.3.4. Diffusion Dialysis for the Mixture of HCl/FeCl_2_

Diffusion dialysis was performed by utilizing the framework discussed in our prior research studies [53,55,56,57,58]. The following is a representation of the typical experimental setup. The tests for the DD were conducted in a two-compartment cell that was segregated via a membrane that had an effective area of about 5.7 cm^2^. All membranes were cautiously stabilized in the feed solution (1 M HCl + 0.25 M FeCl_2_) for two hours prior to the test, which stimulated the waste acid solution generated in industrial processes, for example, metallurgical processes or related products. During the analysis, one compartment of the cell was loaded with 100 mL feed solution and another one with 100 mL distilled water. In an effort to minimize the concentration polarization, both sides were intensely agitated. One hour of diffusion was permitted. Following that, all the solutions, feed, and permeate were extracted from their respective compartments. Titration carried out using Na_2_CO_3_ aqueous solution with a concentration of 0.05 mol/L determined the HCl concentrations on both sides, while titration was performed with KMnO_4_ aqueous solution with a concentration of 0.002 mol/L determined the FeCl_2_ concentration. All of the tests were carried out at a temperature of 25 °C. Using the formula below, the dialysis coefficients (*U*) can be determined [41,48,57]:

(3) $$U=\frac{M}{At\Delta C}$$

where *M* refers to the quantity of the component transferred in (mol) whereas A specifies the effective area of the membrane in (m^2^), t refers to the time (h), and ∆*C* defines the logarithm mean concentration in between the two chambers in (mol/m^3^). ∆*C* is calculated as below [41,48,57]:

(4) $$\Delta C=\frac{C_{f}^{0}-(C_{f}^{t}-C_{d}^{t})}{\ln[C_{f}^{0}/(C_{f}^{t}-C_{d}^{t})]}$$

where $C_{f}^{0}$ and $C_{f}^{t}$ indicate the feed concentrations at time 0 and *t*, respectively, and $C_{d}^{t}$ is the dialysate concentration at time t.

The dialysis coefficients *U_H_* and *U_Fe_* can be evaluated using Equations (3) and (4). The separation factor (*S*) is calculated as the ratio of the dialysis coefficients (*U*) of the two species present in the solution represented in Equation (5) below [41,48,57]:

(5) $$S=\frac{U_{H}}{U_{Fe}}$$

## 3. Results and Discussion

### 3.1. FTIR and Proton NMR Tests

**Figure 7:**
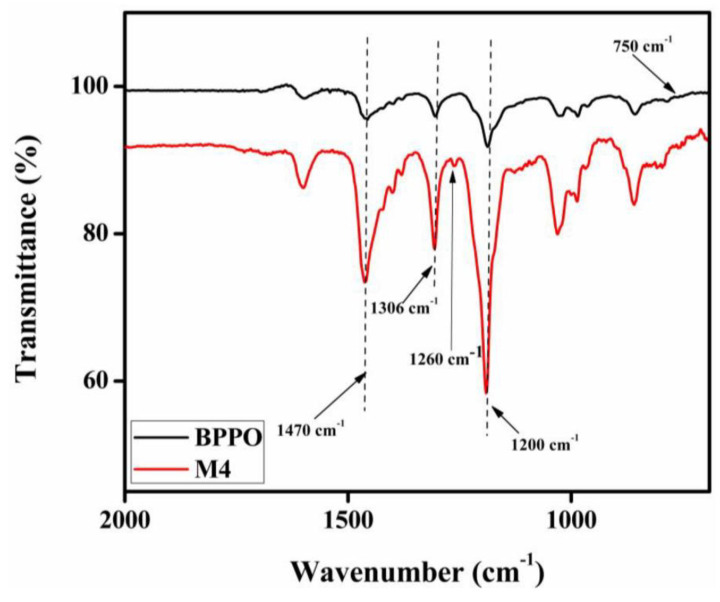
The infrared (IR) spectrum of pristine BPPO and M4 membranes.

The successful formation for TMA functionalized BPPO-based porous membranes is validated through FTIR spectrum analysis. Figure 7 illustrates the FTIR spectra for pristine BPPO as well as the prepared anion exchange membrane. The stretching vibration of –CH groups (V and δ) existing inside the pristine BPPO and also the prepared anion exchange membrane generated the reference band in the range of 1446 cm^−1^ [41,43,52,57]. The symmetrical and asymmetrical stretching vibrations of C-O have adsorption peaks of 1200 cm^−1^ and 1306 cm^−1^, respectively, whilst phenyl groups have peaks of 1470 cm^−1^ and 1600 cm^−1^. The sharp peak observed in the prepared AEM at 1260 cm^−1^ reflects the C-N stretching vibrations, which are missing in the membrane of pristine BPPO. The vibration for C=C stretching present in the phenyl groups is responsible for the band at 1608 cm^−1^, while C-O-C stretching is responsible for the peak at 1190 cm^−1^. [59]. The bands occurring at 1446 cm^−1^ are generated by stretching of –CH groups (V and δ) [43]. The stretching of C-Br in the BPPO membrane caused the band to appear at 750 cm^−1^ [54,60]. This band did not show up in the ATR-FTIR spectra of prepared AEMs after the reaction with TMA. These findings strongly indicate that the prepared AEMs were successfully synthesized.

**Figure 8:**
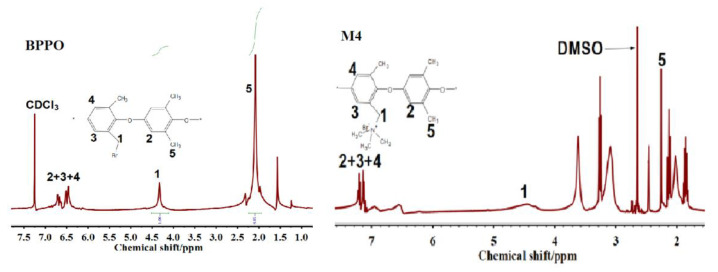
Proton NMR spectrums of BPPO and prepared anion exchange membrane M4.

Moreover, proton NMR spectroscopy was also used to confirm the successful fabrication of anion exchange membranes. Figure 8 depict the proton NMR spectrums of the pure BPPO and fabricated anion exchange membrane M4. After the reaction with trimethylamine, a new peak was observed at 4.5 ppm, which was absent in the proton NMR spectrum of the pure BPPO. This peak is associated with the -CH_2_-N bond into the prepared anion exchange membrane M4, which shows the successful quaternization reaction between BPPO and trimethylamine.

### 3.2. Morphology

**Figure 9:**
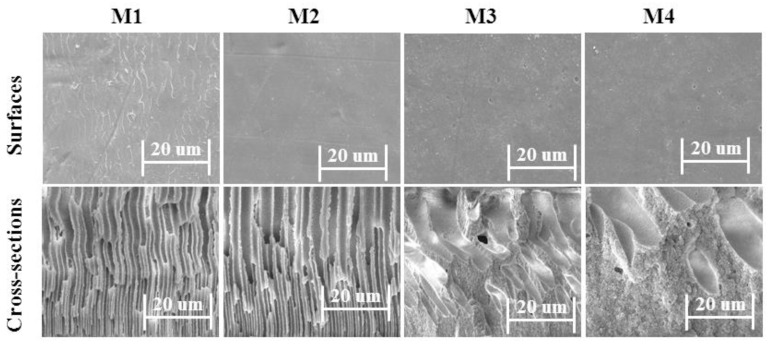
SEM micrographs of surface (**top**) and cross-section (**bottom**) of the prepared porous membranes.

The morphology of the designed membranes was evaluated using scanning electron microscopy (SEM). Figure 9 present the SEM micrograph of the designed membranes’ surface layers and cross-sections. All of the membranes examined were reported to possess a porous morphology. Membrane morphology was determined by the concentration of TMA found in the polymer matrix. The pore size of membranes M1 to M4 increases from 16 μm to 44 μm as the ion exchange group concentration in the polymer matrix increases. Figure 9 show the changes in morphologies of the prepared membranes as the amount of TMA in the membrane matrix was enhanced. In the cross-sectional micrographs of the membranes, pores that have finger-like morphology can be observed. Moreover, Figure 9 clearly show that the presence of finger-like structures in membranes M1 to M4 were increased with the content of amine, which is consistent with our previous findings [41,61]. This can lower the resistance of ions as they pass within the membranes, making it useful for the separation of mixtures. As a result, these porous morphologies may be ideal for acid recovery using the DD process.

### 3.3. Thermal Decomposition and Chemical Stability of the Prepared Membranes

**Figure 10:**
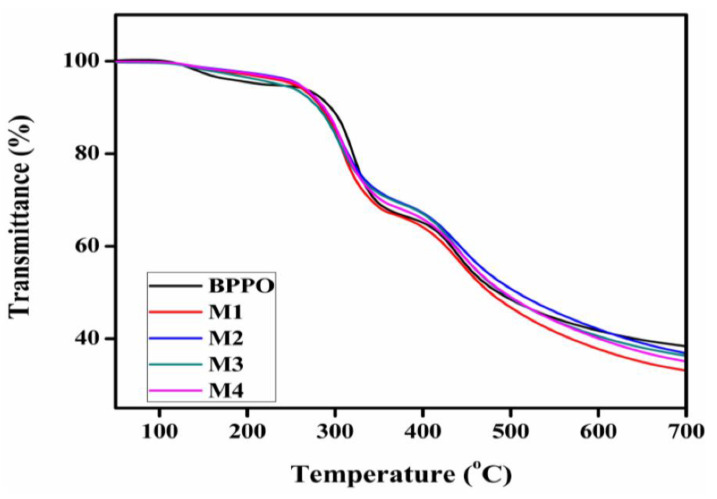
Thermal gravimetric analysis (TGA) thermograms for various porous BPPO-based membranes.

The thermal decomposition behavior for the formulated membranes M1 to M4 is evaluated by TGA, as seen in Figure 10. At medium temperatures, the designed AEMs demonstrate good thermal stability and can withstand temperatures of up to 200 °C. The thermal desorption of water, thermal deamination, and thermal oxidation of the membrane polymer were the three key stages in the weight loss property of the membranes. The vaporization of water from the polymer matrix caused all of the membranes to lose weight between 90–140 °C during the first step. At 250 °C, the quaternary ammonium group deteriorated, culminating in the second weight loss stage [54]. The cleavage of the main polymer matrix is responsible for the final stage of weight loss, which occurred at 420 °C.

**Figure 11:**
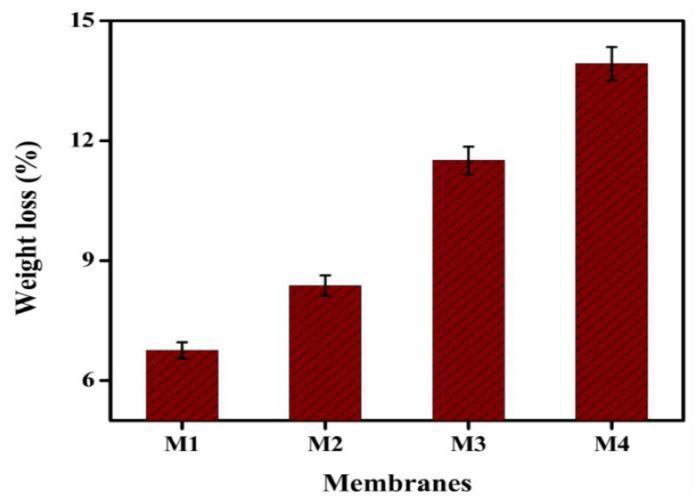
Chemical stability of membrane M1 to M4 after immersion in 2 M HCl for two weeks at room temperature.

Figure 11 demonstrate the chemical stability of the designed AEMs after two weeks at ambient temperature immersion in 2 M concentration of HCl. Here, the weight loss of the membrane after immersion in a 2 M HCl solution at room temperature is referred to as chemical stability. After two weeks of immersion in the 2 M HCl solution, all of the prepared membranes demonstrated good chemical stability, while their color remained unchanged. The weight loss (%) of the prepared membranes ranged from 11.90% to 15.38%, with membranes comprising the most TMA indicated the maximum weight loss. Furthermore, by increasing the amount of TMA in the polymer matrix, weight loss was increased substantially from M1 to M4. Thus, with a two-week immersion, the maximum weight loss was just around 15.38%. This indicated that the AEMs that have been prepared are incredibly effective in terms of DD application for acid recovery.

### 3.4. Ion Exchange Capacity (IEC) and Fixed Group Concentration (C_R_)

A critical variable of AEMs used for the DD process is the ion exchange capacity they have. Hence, this variable was measured by the classical Mohr’s method and depicted in Table 1. The value of IEC was reported in the range of 0.71 mmol/g to 1.43 mmol/g. In other words, it was observed that as the concentration of TMA in the polymer matrix increased, the IEC also enhanced from 0.71 mmol/g to 1.43 mmol/g.

Fixed group concentration (*C_R_*) was typically determined by dividing the IEC (ion exchange capacity) with the *W_R_* (water uptake). According to previous researchers, it is an essential parameter of AEMs, and variations in *C_R_* values can have a significant impact on the DD performance of the membrane [41,48]. At ambient temperature, the *C_R_* (fixed group concentration) of the formulated membranes was evaluated, and the results obtained are presented in Table 1. It was discovered that as the concentration of TMA in the polymer matrix increased, the fixed group concentration (*C_R_*) also surged from 0.0047 mol/L to 0.0056 mol/L.

### 3.5. Water Uptake and Linear Expansion Ratio

IEM’s water uptake (*W_R_*) is a useful tool that has a measurable impact on the phenomena of separation, as well as the dimensional and mechanical characteristics [41,52,62,63,64]. Water molecules existing in the membrane matrix will facilitate the separation of charged functional groups, which is essential for ion transport [52,64]. As presented in Table 1, the *W_R_* (water uptake) of the prepared membranes enhanced from 149.60% to 233.80% as the concentration of TMA in the membrane matrix increased. Capillary tension is primarily responsible for its association with the porous structure. From this perspective, the porous AEMs should possess marginally greater water uptake than dense AEMs whenever the ion exchange capacities are equivalent [41].

IEMs have a significant factor called the linear expansion ratio (*LER*). Membranes with a high *LER* exhibited poor durability and mechanical stability, thereby reducing membrane performance. Table 1 show the findings of the investigation performed at room temperature. It was discovered that increasing the concentration of TMA in the polymer matrix increased the (*LER*) ratio from 3.88% to 9.23%. Furthermore, both water uptake and swelling behavior were influenced by the hydrophilic nature of the membrane matrix as well as the degree of plasticization [65]. The durability of the polymeric matrix ensures an increase in swelling ratio as well as the water uptake at a lower degree of plasticization (i.e., the inclusion of high hydrophilic sites), which is aimed at improving the acid recovery performance [66,67].

### 3.6. Water Contact Angle

**Figure 12:**
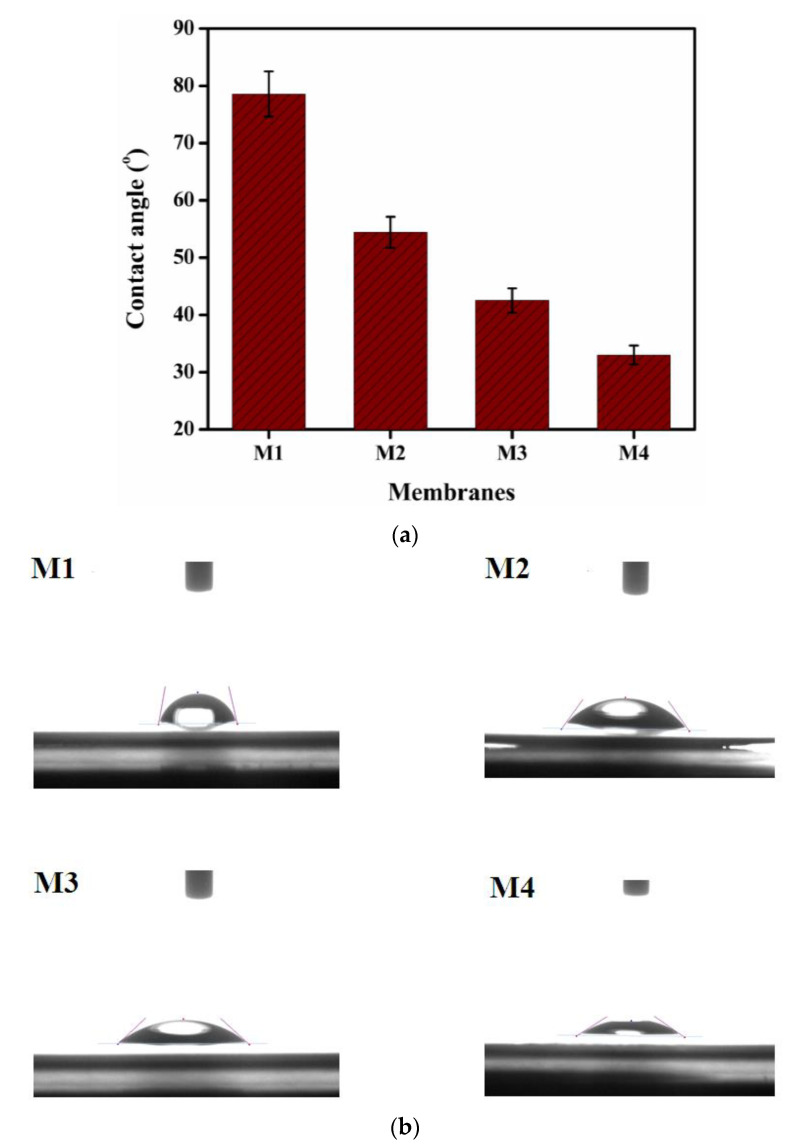
(**a**): water contact angle of membranes M1 to M4; (**b**): Water contact images of membranes M1 to M4.

One of the essential features of ion-exchange membranes is the water contact angle which is used to determine their hydrophilicity. At ambient temperature, the water contact angle of prepared membranes M1 to M4 was determined, and the results are shown in Figure 12a,b. The water contact angle for the formulated membranes was observed to be lowered from membranes M1 to M4 as the concentration of TMA in the membrane matrix increases, as per these figures. This is related to the enhancement in the hydrophilic nature of membranes as the ion exchange contents in the polymer matrix increased. For DD applications, the increased hydrophilicity of prepared membranes is favorable.

### 3.7. DD for HCl/FeCl_2_ Solution

The acid dialysis coefficient (*U_H_*) and acid/salt separation factor (*S*) of AEMs are employed to directly assess the ability of recovery and purity for the acid that is recovered; these are the essential factors to evaluate DD efficiency. Nevertheless, certain commercial AEMs have low acid recovery ability during the DD process, which restricts their use in acid recovery applications. Due to the extreme practical implementation of AEMs in DD, the developed AEMs with better acid dialysis coefficients must be designed based on the structure–performance relationship of membrane materials [23].

**Figure 13:**
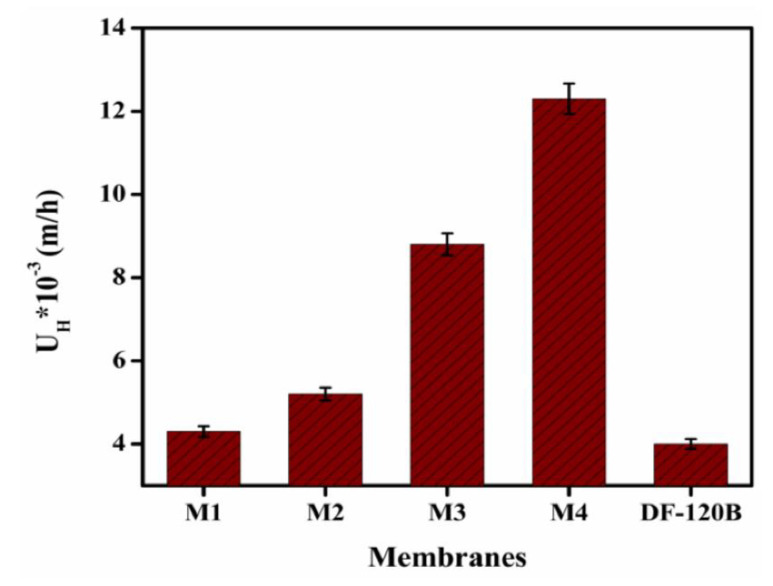
Acid dialysis coefficient (*U_H_*) at 25 °C for different representative porous BPPO-based membranes.

After the in-depth analysis and comprehensive characterizations of the prepared porous membranes, an experiment was conducted in this study using an HCl + FeCl_2_ mixture with concentrations of 0.1 M HCl and 0.25 M FeCl_2_ for exploring possible applications for the membrane applications in acid recovery through diffusion dialysis process. According to the obtained DD results, it was found that the dialysis coefficient of HCl (*U_H_*) for the prepared membranes ranges from 0.0043 m/h to 0.012 m/h at room temperature, as presented in Figure 13. It was also discovered that as the concentration of TMA in the polymer matrix increased, the values of *U_H_* gradually increased from membranes M1 to M4. As a result, based on the aforementioned findings, two separate references may be obtained. Primarily, the recorded *U_H_* values are relatively larger than those of commercial DF-120B membrane with *U_H_* value of 0.004 m/h. Secondly, these values are nearly identical to our formerly published membranes with *U_H_* around 0.010 m/h at a temperature of 25 °C [41]. The porous structure and growth in the ion-exchange group inside the membrane matrix may indeed be primarily responsible for the steady increase in *U_H_* values. The active zone, also known as the ion-exchange party, is present in all the prepared porous membranes and is responsible for ion transfer across the membrane. As the amount of TMA in the polymer matrix was gradually increased from membrane M1 to M4, the IEC and *W_R_* of the membranes also increased, resulting in enhanced hydrophilicity of membranes and higher *U_H_* values of the prepared membranes. The presence of the –N^+^(CH_3_)_3_Br^−^ group in the membranes significantly enabled Cl^−^ ions to move through them. H^+^ ions can also pass across the membranes with Cl^−^ to achieve electrical neutrality [68]. Fe-related components such as Fe^2+^ and FeCl^+^, on the other hand, are less likely to move through the membrane due to their greater size and reduced mobility [67,68].

**Figure 14:**
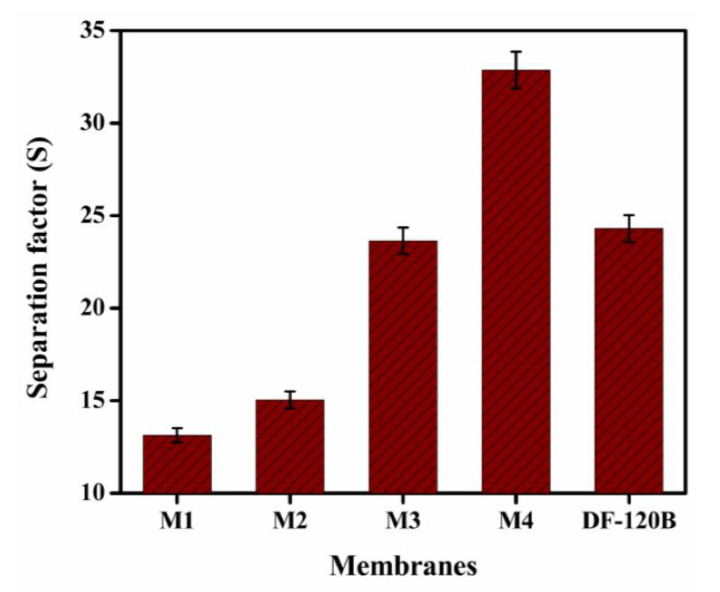
Separation Factor at 25 °C for multiple representatives for porous BPPO-based membranes.

Figure 14 depict the values of separation factor (*S*) as a ratio of *U_H_* to *U_Fe_*. The S values acquired for the prepared membrane M1 to M4 range from 13.14 to 32.87, implying that the S value for the prepared membrane M4 is almost identical to that of PVA-based hybrid membranes (22–39) at 25 °C [63], (12.1–35.7) at 25 °C [43], and (18.5–21) at 25 °C [69]. Previous studies have indicated that the separation factor is influenced by the structure of the membrane and its functional groups. This study indicated that as the amount of TMA in the prepared membranes M1 to M4 increases, the IEC values gradually increase and that this increased IEC is effective in finding a high S value. Furthermore, as shown by the SEM micrographs in Figure 9, the prepared membranes M1 to M4 contain porous morphologies. During the ion transport process, the resistance was reduced by the porous morphology. Because the membrane structure is porous, H^+^ ions can transfer easily, increasing the *U_H_* value; however, smaller pore sizes can greatly impede the transport of FeCl_2_, increasing the *S* values. Hence, from the prepared membranes M1 to M4, we see a rising trend in *S* values. The prepared porous membranes M1 to M4 can be effectively used for acid recovery through diffusion dialysis process from the perspective of acid dialysis coefficient and separation factor.

## 4. Conclusions

Porous BPPO-based AEMs were developed using a phase-inversion technique in ethanol medium in this manuscript. The trimethylammonium group in the prepared AEMs was responsible for Cl^−^ transfer within the polymer matrix. FTIR and proton NMR tests verified the reaction between BPPO and TMA. The thermal and acid stability of the prepared membranes were exceptional. Additionally, the prepared membranes were proven to have a higher *W_R_* (water uptake), IEC (ion exchange capacity), and *C_R_* (fixed group concentration). The increased hydrophilicity of the developed membranes was demonstrated by the water contact angle. The DD process for the recovery of acid was used to investigate the separation efficiency of the prepared membranes at ambient temperature. The observations led to a higher dialysis coefficient of acid (HCl) (0.0034–0.012 m/h) and a higher separation factor (*S*) (13.14–32.87). Therefore, diffusion dialysis can be potentially applied to recover acid using these formulated porous BPPO-based anion exchange membranes.

## Figures and Tables

**Table 1:** Composition, ion exchange capacity, fixed group concentration, water uptake, and membranes swelling of membranes M1 to M4.

Sr. No	BPPO (g)	TMA (g)	IEC (mmol/g)	*W_R_* (%)	*LER* (%)	C × 10^−3^ (mol/L)
M1	3	0.15	0.71	149.60	3.88	4.74
M2	3	0.20	0.98	170.45	5.0	5.75
M3	3	0.25	1.20	196.42	5.98	6.10
M4	3	0.30	1.43	233.80	9.23	6.12

## Data Availability

Not applicable.

## Abbreviations

AEM	Anion exchange membrane
BPPO	Brominated poly(2,6-dimethyl-1,4-phenylene oxide)
CR	Fixed group concentration
DD	Diffusion dialysis
IEC	Ion exchange capacity
IEM	Ion exchange membrane
NMP	N-Methyl-2-pyrrolidone
SEM	Scanning electron microscopy
S	Separation factor
TGA	Thermogravimetric analysis
TMA	Trimethylamine
UH	Diffusion dialysis coefficient of HCl
UFe	Diffusion dialysis coefficient of FeCl2
WR	Water uptake
